# Rapid screening of riot control agents using DART-TD-HRMS

**DOI:** 10.1007/s11419-024-00681-5

**Published:** 2024-02-22

**Authors:** Lina Mörén, Anders Östin, Andreas Larsson, Julia Forsberg, Daniel Wiktelius, Pernilla Lindén

**Affiliations:** https://ror.org/0470cgs30 grid.417839.0 0000 0001 0942 6030 FOI, Swedish Defence Research Agency, CBRN Defence & Security, SE 901 82 Umeå, Sweden

**Keywords:** DART-TD-HRMS, Riot Control Agent (RCA), Chemical Weapons Convention (CWC), Self-defence sprays, High throughput screening

## Abstract

**Purpose:**

Riot Control Agents (RCAs) are chemicals used in law enforcement for non-lethal riot control and use in conflicts between states that violates the Chemical Weapons Convention. OPCW's Scientific Advisory Board has identified sixteen potential RCAs including capsaicinoids, CS, and CR. RCAs may be misused for criminal purposes, so methods for detecting such misuse are needed. This study therefore evaluates the feasibility of a rapid, high throughput screening method of RCAs on surfaces (particularly clothing surfaces) by Direct Analysis in Real Time with a thermal desorption unit coupled to high-resolution mass spectrometry (DART-TD-HRMS).

**Methods:**

A broadly applicable method for detecting potential RCAs was developed and tested on cotton fabric samples sprayed with self-defence sprays from an in-house reference stock. The feasibility of detecting RCAs by direct analysis of surface wipe samples placed in the DART source was also investigated.

**Results:**

The method detected all sixteen RCAs and contaminated clothing were successfully screened for active agents in a reference collection of self-defence sprays. A pilot study also showed that RCAs can be detected by holding a sample directly in front of the DART source.

**Conclusion:**

DART-TD-HRMS enables rapid and simple screening of RCAs on fabric samples enabling a high sample throughput.

**Supplementary Information:**

The online version contains supplementary material available at 10.1007/s11419-024-00681-5.

## Introduction

Riot Control Agents (RCA) are substances that were developed to enable law enforcement officers to temporarily incapacitate people in riot situations [1]. RCAs interact with sensory nerve receptors to induce local discomfort and/or pain together with consequential reflexes that temporarily disable the subject [2]. The Chemical Weapons Convention (CWC) prohibits the use of chemical agents (including RCAs) in military conflicts and requires each State Party to provide a declaration containing a comprehensive list of chemicals held for the purpose of riot control [3, 4]. The Organisation for the Prohibition of Chemical Weapons (OPCW) lists sixteen substances with potential to be used as RCAs, which are listed in Table 1 [5]. The substances most commonly used as RCAs at present are oleoresin capsicum (OC) extracts, 2-chlorobenzalmalononitrile (CS), and dibenzoxazepine (CR) [2, 6]. 2-Chloroacetophenone (CN) was frequently used historically by organizations including the U.S. Army but has largely been replaced by CS, which is considered to be both less toxic and more potent [7, 8].

**Table 1:**
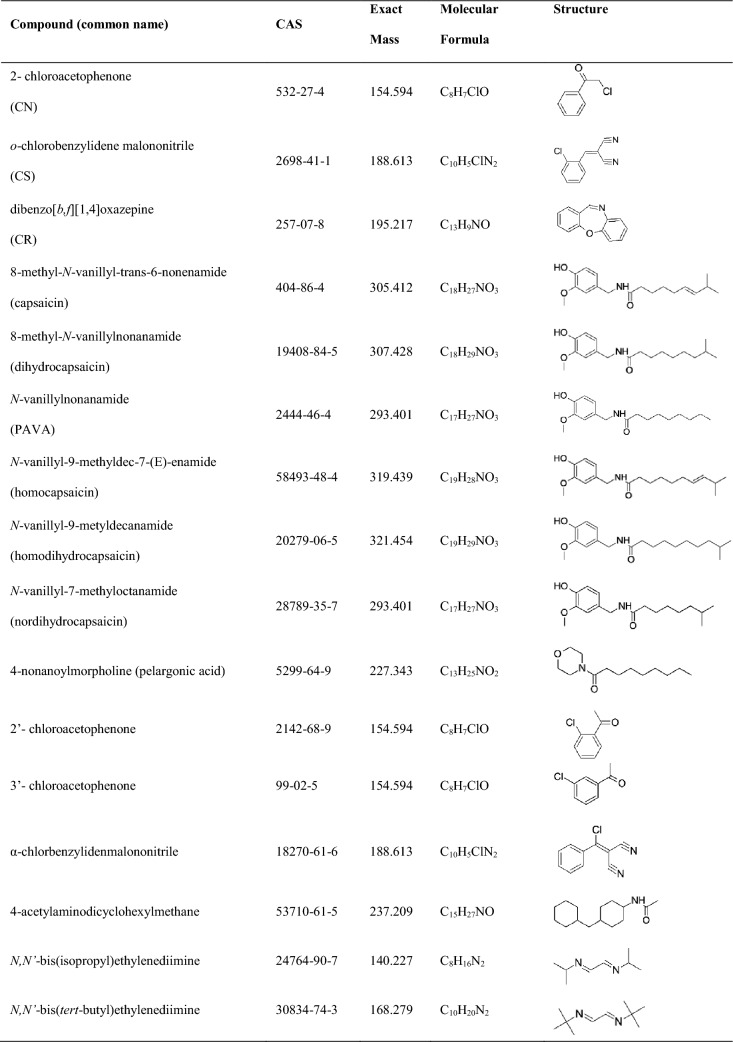
Potential active substances in modern RCAs according to the OPCW Scientific Working Group [38]

There is a wide range of devices for dispersing RCAs, ranging from hand-held self-defence spray cans that generate aerosols to grenades that disperse agents using pyro-techniques [7]. This work focuses on handheld self-defence sprays, which are sometimes informally referred to as pepper spray. Pepper spray is named after the chilli pepper plant from which OC is extracted [9]. Here we refer to RCA-containing handheld spray canisters as *self-defence sprays*. The most common active agents in self-defence sprays are OC and CS, both of which are solids at room temperature and must therefore be suspended or dissolved in a carrier before they can be aerosolised. Handheld sprays also typically contain a propellant to facilitate dispersion. The spray may be a fine mist, liquid, gel, or foam stream [10]. Here we refer to both carriers and propellants as additives. Self-defence sprays generally contain mixtures of additives including butyl diglycol, ethanol, propylene glycol, dipropylene glycol, isopropanol, and 2,2-dimethoxypropane [10, 11].

While self-defence sprays are legal for self-protection in some EU countries including the Czech Republic, Spain, and Poland [12–14], they are regulated in others such as Sweden and Belgium [15, 16]. In the UK, pepper sprays are fully banned [17]. Self-defence sprays have been misused for antagonistic or criminal acts. A severe example occurred in Turin in 2018, where a canister containing an RCA was shot into a crowded city square to facilitate the theft of valuable property during the arising chaos. The resulting panic caused over 1500 people to suffer injuries and led to one fatality [18, 19].

When a canister is discovered or seized at an incident site, standard protocols require that it be sent to a laboratory for further analysis and identification. When the source of a potential RCA (e.g., a canister) is available, many different analytical strategies can be applied; active substances in seized self-defence spray canisters can be detected by gas chromatography (GC) and liquid chromatography (LC) mass spectrometry (MS) or by Direct Analysis in Real Time (DART-MS) [11, 20–23]. However, Swedish Hazmat teams frequently handle minor incidents involving the dispersal of irritating chemicals where pepper spray or tear gas are suspected to have been used but no canister is present for analysis. There are reports describing the identification of capsaicinoids on fabric samples by both GC- and LC–MS, but these methods require extensive sample preparation and long analysis times [10, 24]. Consequently, they are unsuitable in cases where the irritating chemical must be identified rapidly by surface sampling to implement effective countermeasures. Unfortunately, dispersed compounds with low volatility are difficult to detect and analyse in the field, necessitating the development of protocols for efficient sampling and transport to a nearby laboratory that can rapidly analyse and identify any RCAs that are present. Depending on the incident, it is also possible that a large number of samples are collected and need to be screened for content. It is for example difficult to sample large the target materials on the surfaces for further analysis by GC- and LC–MS. However, using sample traps that can be rapidly analysed by DART-MS as a first high throughput screening method, a contaminated area can be located and further sampled and analysed with other confirmatory methods.

We have therefore developed a method for fast analysis of RCAs using a DART source with a thermal desorption (TD) unit coupled to a high-resolution mass spectrometry (HRMS) instrument. The DART ionization technique is commonly used in forensic applications as a screening tool that can provide confirmatory results [25–27]. Both CS and capsaicin have previously been analysed by the DART AccuTOF detector [20]. In these systems, the TD module heats the sample prior to mass detection, improving reproducibility and allowing simple sample introduction [28]. Moreover, the high mass accuracy of the HRMS enables mass-based separation of simultaneously detected compounds [29].

The first step in the method’s development was to establish a reliable protocol for detecting the sixteen potential RCAs listed by the OPCW [5]. To evaluate the linearity of the signal intensity with respect to the amount of analyte presented to the detector, concentration curves were established for selected compounds.

We then evaluated the method’s ability to identify unknown and potentially hazardous compounds from incidents in which people show symptoms suggesting the presence of an irritant. To this end, twenty self-defence sprays from an in-house reference collection were dispersed on cotton fabric and sampled by wiping a sample trap over the fabric surface. Finally, we conducted a short pilot study to evaluate the possibility to analyse samples placed directly between the DART source and the MS detector without using a TD unit. Two types of surface samples were tested: cotton fabric sprayed with self-defence spray and cotton swabs or wipes that were wiped over ceramic floor tiles sprayed with self-defence spray. Our results show that DART-MS is an attractive technique for analysing such samples because it offers a short analysis time, requires no sample preparation and enables a high sample throughput.

## Material and methods

### Chemicals and self-defence sprays

All chemicals were purchased at the highest available purity. 2-Chloroacetophenon (product no. C19686), *N*-vanillylnonamide (PAVA, V9130-1G), 2ʹ-chloroacetophenone (product no. 183709-25G), 3ʹ-chloroacetophenone (product no. 288799-5G) and dihydrocapsaicin (product no. 03813-5MG) were purchased from Sigma Aldrich (ST Louis, MO, USA). Other purchased compounds were capsaicin from UPS reference standard (US Pharmacopeia, Rockville, MD, USA). 2-Chloro(phenyl)methylene)malononitrile (α-chlorobenzylidenmalononitrile) from Ambeed (Arington Hts, IL, USA), and *N*-nonanoylmorpholine (4-nonanoylmorpholine) (product no. 341991, Fluorochem Ltd, Hadfield, UK). The following reference compounds were synthesized in-house: *N,N'*-bis(tert-butyl)ethylenediimine and nordihydrocapsaicin as described by Wiktelius et al. [30]. CS, CR, homocapsaicin, homodihydrocapsaicin, c*is*-4-acetylaminodicyclohexylmethane, *N,N'*-bis(isopropyl) ethylenediimine (see Supplementary Materials, Data S1 for details of their synthesis). The acetonitrile used was hyper grade for LC–MS LiChrosolv from Supelco (cat. no 1.00029.2500, Merck KGaA, Darmstadt, Germany). The self-defence sprays included in the study were in-house reference materials obtained from vendors who reported their active agents to be either OC or CS. Some of these sprays were as much as 4 years past their expiry date (Supplementary Materials, Table S1).

### Sample preparation

The sixteen RCA compounds were dissolved in acetonitrile (ACN) and diluted with ACN to predefined concentrations. Concentration curves were established for each RCA by applying 5 µl aliquots with concentrations of 0.1, 0.2, 1, 2, 5, 10, 20, and 100 ng/µl to the sample trap (DSA Detection, North Andover, MA, USA, part no: ST1318P), consisting of a special swab in glass fibre developed to fit the TD unit), and letting them dry before performing DART-TD-HRMS analysis (the drying time was typically 3–5 min). A photograph of the DART-TD-HRMS setup and a sample trap can be found in Supplementary Material, Fig S1. Reproducibility was evaluated by applying 5 µl of a 10 ng/µl solution of the compound to seven sample traps and letting it dry before analysis. Sample traps were analysed in series. For *N,N'*-bis(isopropyl)ethylenediimine and *N,N'*-bis(tert-butyl)ethylenediimine 5 µl of a 100 ng/µl solution was used. Robustness was evaluated by analysing 5 µl aliquots of each compound at a single concentration over three consecutive days; as before, the traps were allowed to dry before analysis by DART-TD-HRMS. The limit of detection (LOD) for CS, CR, capsaicin and PAVA was determined by analysing serial dilutions of these compounds. The lower limit of detection (LLOD) was defined as the lowest concentration giving a peak with a signal-to-noise (S/N) ratio above 10 and the upper limit was the highest concentration tested before the detector became saturated.

Twenty commercial self-defence sprays were sprayed, with a distance of 10–15 cm, onto pieces of cotton fabric (Ohlssons Tyger & Stuvar AB, Umeå, Sweden) measuring 15 × 15 cm, placed in a fume hood. Five of the sprays had CS as the active substance, five had PAVA, and ten had capsaicin. The sprays were not all equally efficient; short bursts of roughly 1–2 s produced different-sized stains. Some sprays were coloured, making them easy to see and sample, while others were colourless and harder to sample with precision once the stain had dried. Surface sampling of the fabric was done by wiping a sample trap once over the stain when sampling 1 h and 1 week after spraying, and by wiping twice, sampling 2 or 3 weeks after spraying.

An additional pilot study was done to evaluate the feasibility of analysing self-defence sprays without using the desorption unit. Two different sprays were used; in each case, 100 µl of undiluted spray was applied to a piece of cotton (3 × 3 cm) and an additional 100 µl of spray was applied directly to a ceramic floor tile. The cotton fabric was held directly between the DART source and the MS inlet for a few seconds, while the ceramic floor tile was sampled using a dry cotton swab or a cotton wipe that was then held between the DART source and the MS inlet for a few seconds.

### DART-TD-HRMS

Samples were analysed using a Direct Analysis in Real Time-Simplified Voltage and Pressure (DART-SVP) ion source (IonSense Inc., Saugus, MA, USA) with a Vapur®-interface (SI-410-GIST, IonSense Inc., Saugus, MA, USA) and a Thermal Desorption unit (Bruker-IonSense Inc., Saugus, MA, USA). Analytes were detected using a Maxis Impact time-of-flight high-resolution mass spectrometer (TOF-HRMS; Bruker Daltonics, Bremen, Germany). The mass spectrometer was calibrated with the ESI ion source, a syringe pump and a calibration standard (LC/MS Calibration standard, for ESI-TOF, 100 ml, part number G1969-85,000, Agilent Technologies, Santa Clara, USA) following the procedure stated by the manufacturer, prior to mounting the DART interface. Nitrogen was used as the DART ionization gas. Samples were introduced to the thermal desorber using sample traps. The operating temperatures of both the DART source and the thermal desorption unit were set to 300ºC. The DART ion source was run in positive mode with a grid voltage of 350 V. The mass spectrometer was run in positive mode using full scan and broadband collision-induced detection (bbCID) (except the self-defence sprays which were analysed with full scan only) with a mass range of 50–650 *m/*z at a frequency of 5 Hz; fragments were verified by MS/HRMS at 25 eV.

### Data processing and analysis

Data processing and analysis were done using DataAnalysis 4.2 (Bruker Daltonik GmbH, Bremen Germany). A compound was defined as found if the [M + H]^+^ ion had a S/N > 10. Self-defence sprays were screened for six compounds: capsaicin *m/z* 306.2064, PAVA *m/z* 294.2064, dihydrocapsaicin *m/z* 308.2220, homocapsaicin *m/z* 320.2220, homodihydrocapsaicin *m/z* 322.2377, and CS *m/z* 189.0214. Molecular formula prediction was examined using the “Smart Formula” tool that is built into DataAnalysis 4.2 (Bruker Daltonik GmbH); the suggested formula whose mass error most closely matched that of the correctly predicted agent in the spray under investigation was used.

## Results

### Method development for detection of potential RCAs

**Fig. 1:**
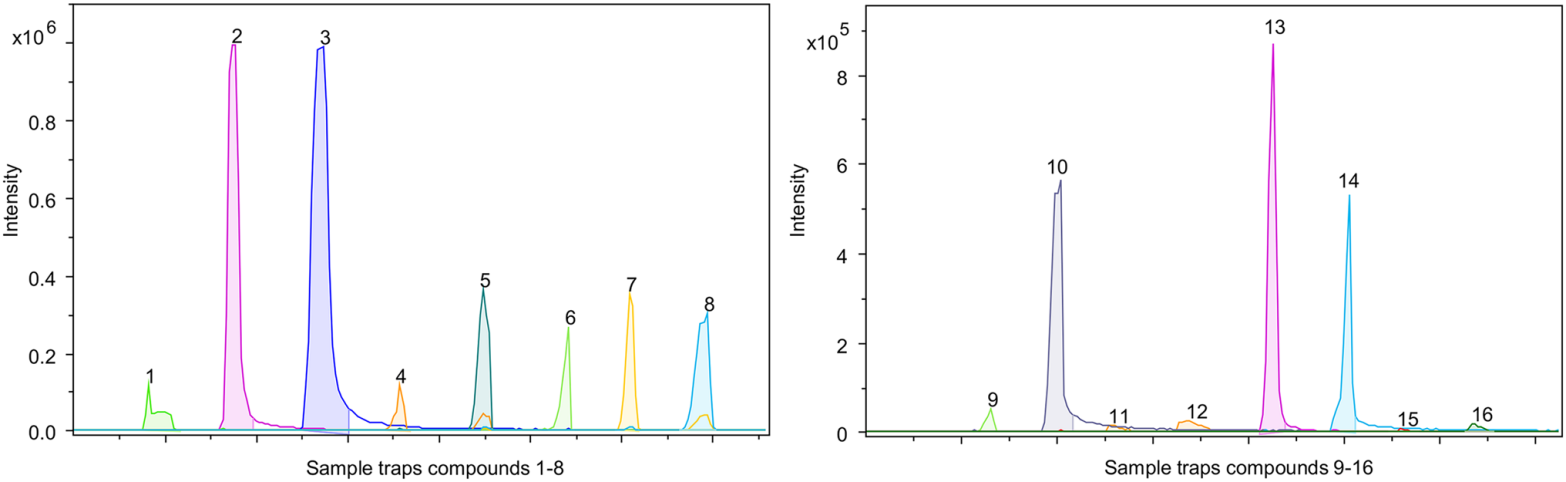
The presented DART-TD-HRMS method could detect all sixteen compounds listed as potential RCAs by the OPCW. All compounds were analysed at 50 ng on the sample strip of compound except compound 15 and 16 which required 500 ng on the sample strip. Chronogram of all compounds, in order as numbered in figure; (1) CN, (2) CS, (3) CR, (4) capsaicin, (5) dihydrocapsaicin, (6) PAVA, (7) homocapsaicin, (8) homodihydrocapsaicin, (9) nordihydrocapsaicin, (10) 4-nonanoylmorpholine, (11) 2ʹ-chloroacetophenone, 12) 3ʹ-chloroacetophenone, (13) α-chlorbenzylidenmalononitrile, 14) *cis*-4-acetylaminodicyclohexylmethane, 15) *N,N*ʹ-bis(isopropyl)ethylenediimine, 16) *N,N*ʹ-bis(tert-butyl)ethylenediimine

A DART-TD-HRMS method was developed with the aim of detecting all sixteen potential RCAs listed by the OPCW, which are listed in Table 1 [5]. Each RCA was dissolved in ACN at different concentrations and droplets of the resulting solutions were either placed on the sample trap and allowed to dry or placed on a surface that was then wiped with the sample trap. The trap was then analysed directly by DART-TD-HRMS. Four parameters were selected for optimization: the applied sample volume, the sample concentration, and the DART source and TD temperatures. The most important parameters for detection were the TD and DART source temperatures, and the best results were achieved when both of these temperatures were set to 300°C, which is 50ºC above the recommended upper operating limit of the TD and the highest temperature tested. Notably, it is also 50°C below the default temperature of the DART source. While these settings were not optimal for every tested compound, they represented a good compromise that enabled the detection of all sixteen target analytes. Using the optimized method with 50 ng of analyte, all sixteen compounds were detected with an S/N > 10 with the exception of *N,N'*-bis(isopropyl)ethylenediimine and *N,N'*-bis(*tert*-butyl)ethylenediimine 500 ng of material (5 µl of a 100 ng/µl solution) was needed to reach the same S/N (Fig. 1).

One concern with the DART technique is the reproducibility and robustness of the analysis. It quickly became apparent that the volume of solution placed on the sample trap greatly affected reproducibility; higher RSD values were obtained when applying 10 µl droplets than when using 5 µl droplets (data not shown), so all subsequent experiments were performed using 5 µl sample volumes. The method’s reproducibility was then evaluated by analysing seven replicate samples of each compound at trap loadings of 50, 100, and 500 ng. Eight of the compounds had RSD values below 20%, while the RSDs for the other eight ranged from the low twenties to 38% in the case of nordihydrocapsaicin. With the exception of PAVA (RSD 10%), the OCs generally had higher RSDs than the other analytes (Supplementary Materials, Table S2).

The robustness of the DART measurements was determined by analysing 50 ng of each compound over 3 consecutive days (Supplementary Materials, Table S2). The technique was most robust for CS, CR, 4-nonanoylmorpholine, α-chlorobenzylidenemalononitrile, and *cis*-4-acetylaminodicyclohexylmethane, which all had RSDs below 20%. Seven compounds had RSDs of 50% or higher, and the RSD for *N,N'*-bis(tert-butyl)ethylenediimine was as high as 113%.

The method’s linearity and LOD were examined for CS, CR, capsaicin and PAVA. The LLOD values based on the S/N > 10 criterion was 5 ng for all four compounds; CS and CR were detected even when using only 1 ng and 0.5 ng, respectively, but with an S/N below ten. The upper limit (i.e., the highest analyte mass at which the detector showed no signs of saturation) was 500 ng for capsaicin and PAVA and 100 ng for CS and CR. CS, CR and capsaicin had detection between 5 and 100 ng while CR had detection between 5 and 50 ng (Supplementary Material, Fig S2).

Seven compounds on the OPCW list had identical mass with at least one other compound. CN, 2’-chloroacetophenone, and 3’-chloroacetophenone all have the same exact mass (154.019 Da), as do CS and α-chlorobenzylidenemalononitrile (188.014 Da) and PAVA and nordihydrocapsaicin (293.199 Da). Since no chromatographic separation was performed before ionization, the only option left to distinguish between them was potential differences in fragmentation pattern. This was the case for CS and α-chlorobenzylidenemalononitrile, where CS had a unique fragment in the bbCID spectra, *m/z* 162. 0166, probably due to the loss of its two nitrogen’s. This fragment ion was not present in α-chlorobenzylidenemalononitrile (mass spectra provided in the Supplementary Materials, Fig S3). PAVA and nordihydrocapsaicin showed identical fragmentation patterns and could not be distinguished from each other. CN, 2’-chloroacetophenone, and 3’-chloroacetophenone all had the same fragmentation pattern, though CN ionized considerably better than the other two. If separation is essential, a complementary analysis using a chromatographic method like GC- or LC–MS should be performed.

### Analysis of self-defence sprays on clothing

The method developed for the OPCW compounds was assessed by analysing clothing samples exposed to twenty commercial self-defence sprays. A burst of spray was applied to a piece of cotton fabric to simulate a real-life scenario in which an RCA is suspected to be present on clothing. One hour after spraying, the fabric was sampled using a sample trap and analysed directly by DART-TD-HRMS to detect active agents (CS, CR and OC) as well as other relevant compounds from the list (i.e., the OC family members dihydrocapsaicin, homocapsaicin, and homodihydrocapsaicin). The active substances stated on the spray cans were detected on all fabrics analysed 1 h after spraying (Table 2). The sprays with capsaicin or OC-capsaicin as the active agent also contained the OCs dihydrocapsaicin, homocapsaicin, and homodihydrocapsaicin.

**Table 2:** Stated and detected active substance in twenty self-defence sprays analysed by DART-TD-HRMS. Each spray was applied to pieces of cotton that were then sampled using sample traps one hour after application

ID	Stated Active Substance	Detected Active Substance	Other OCs detected
Spray 1	OC-PAVA	PAVA	Capsaicin, dihydrocapsaicin
Spray 2	OC- capsaicin	capsaicin	Dihydrocapsaicin, PAVA, homocapsaicin, Homodihydrocapsaicin
Spray 3	OC- capsaicin	capsaicin	Dihydrocapsaicin, PAVA, homocapsaicin, Homodihydrocapsaicin
Spray 4	CS	CS	None
Spray 5	CS	CS	None
Spray 6	OC- capsaicin	capsaicin	Dihydrocapsaicin, PAVA, homocapsaicin, Homodihydrocapsaicin
Spray 7	OC-PAVA	PAVA	Capsaicin
Spray 8	OC-PAVA	PAVA	Capsaicin, dihydrocapsaicin,
Spray 9	OC- capsaicin	capsaicin	Dihydrocapsaicin, PAVA, homocapsaicin, Homodihydrocapsaicin
Spray 10	CS	CS	None
Spray 11	OC- capsaicin	capsaicin	Dihydrocapsaicin, PAVA, homocapsaicin, homodihydrocapsaicin
Spray 12	OC- capsaicin	capsaicin	Dihydrocapsaicin, PAVA, homocapsaicin, homodihydrocapsaicin
Spray 13	CS	CS	none
Spray 14	OC-PAVA	PAVA	Capsaicin, dihydrocapsaicin
Spray 15	OC- capsaicin	capsaicin	Dihydrocapsaicin, PAVA, homocapsaicin, Homodihydrocapsaicin
Spray 16	OC- capsaicin	capsaicin	Dihydrocapsaicin, PAVA, homocapsaicin, Homodihydrocapsaicin
Spray 17	OC- capsaicin	capsaicin	Dihydrocapsaicin, PAVA, homocapsaicin, Homodihydrocapsaicin
Spray 18	OC-capsaicin	capsaicin	Dihydrocapsaicin, PAVA, homocapsaicin, Homodihydrocapsaicin
Spray 19	OC-PAVA	PAVA	None
Spray 20	CS	CS	None

The additives reported on the self-defence spray cans were isopropanol, dipropylene glycol, propylene glycol, ethanol, and glycerol (Supplementary Materials, Table S3). These additives were therefore also analysed. Ethanol and isopropanol could not be detected because of their low molecular masses but the other three additives were detectable; dipropylene glycol ([M + H]^+^ 135.1016 and ([2M + H]^+^ 269.1959) was usually the most abundant of the four. In addition, butyl diglycol ([M + H]^+^ 163.1329) and butyl acetate ([M + H]^+^ 117.0910) were detected and there were two highly abundant peaks that could not be identified despite obtaining predicted molecular formulas; these unknown analytes were designated unknown C_6_H_10_O_2_ ([M + H]^+^ 115.0772) and unknown C_7_H_13_N_4_ ([M + H]^+^ 153.1135).

**Fig. 2:**
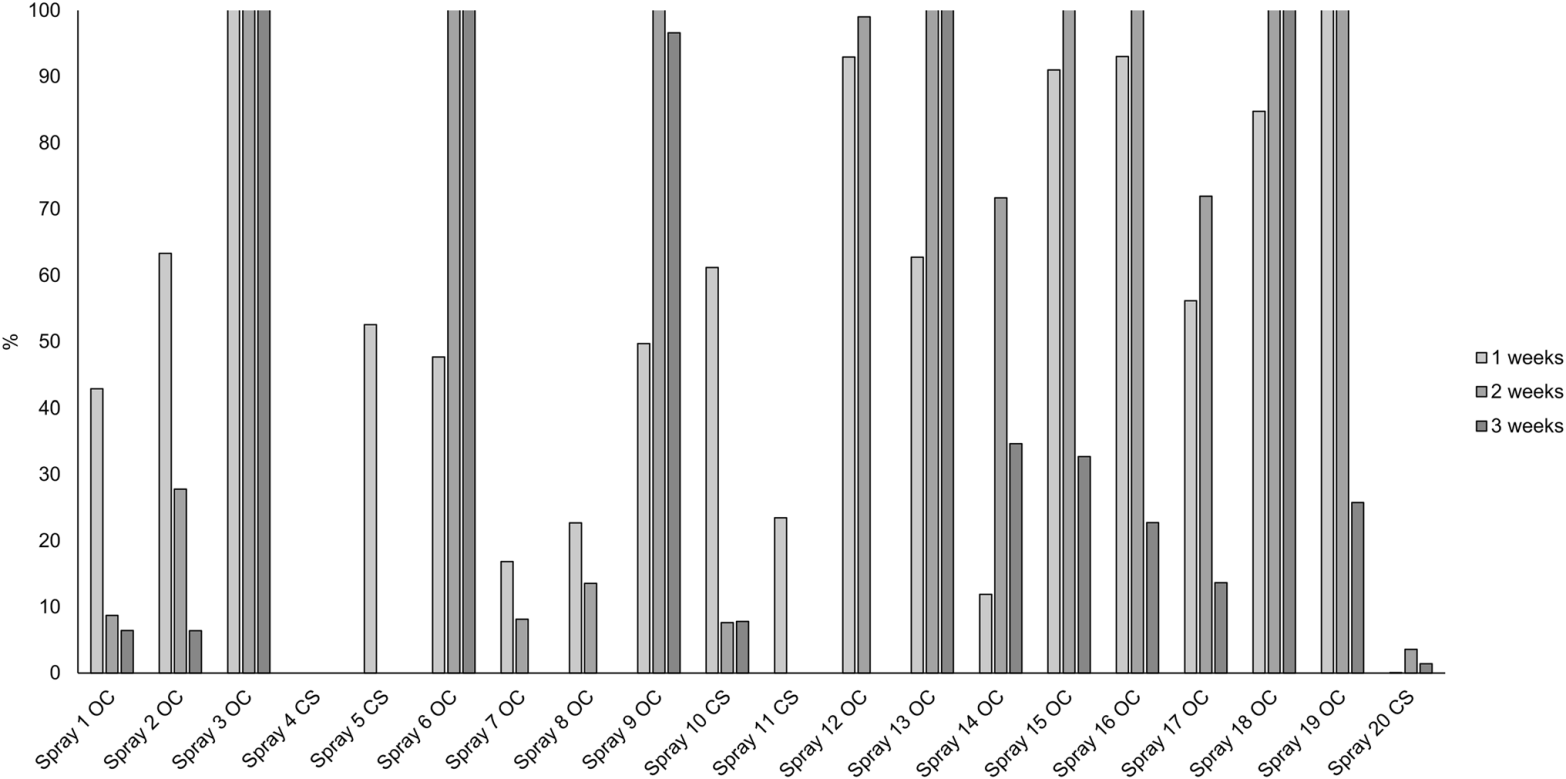
Detected contents of active agents on cotton fabric 1, 2, and 3 weeks after spraying relative to those observed 1 h after spray application. The self-defence sprays contain high quantities of volatile additives giving rise to ion suppression. As the additives evaporate over time, the suppression issue diminishes and the signal of the active substance can therefore be higher 1 week after spraying compared to 1 h after spraying

The pieces of fabric were left in a fume hood and sampled again 1, 2, and 3 weeks after spraying using the same sampling procedure. Figure 2 shows the proportion of the active substance (OC or CS) that was detected on the fabric in each case relative to the results obtained 1 hour after spraying. All CS sprays but one were detected after 1 week; after 3 weeks, all of the OC sprays and two of the CS sprays were detected. We found no correlation between the sprays’ expiry dates and their active agent contents.

**Fig. 3:**
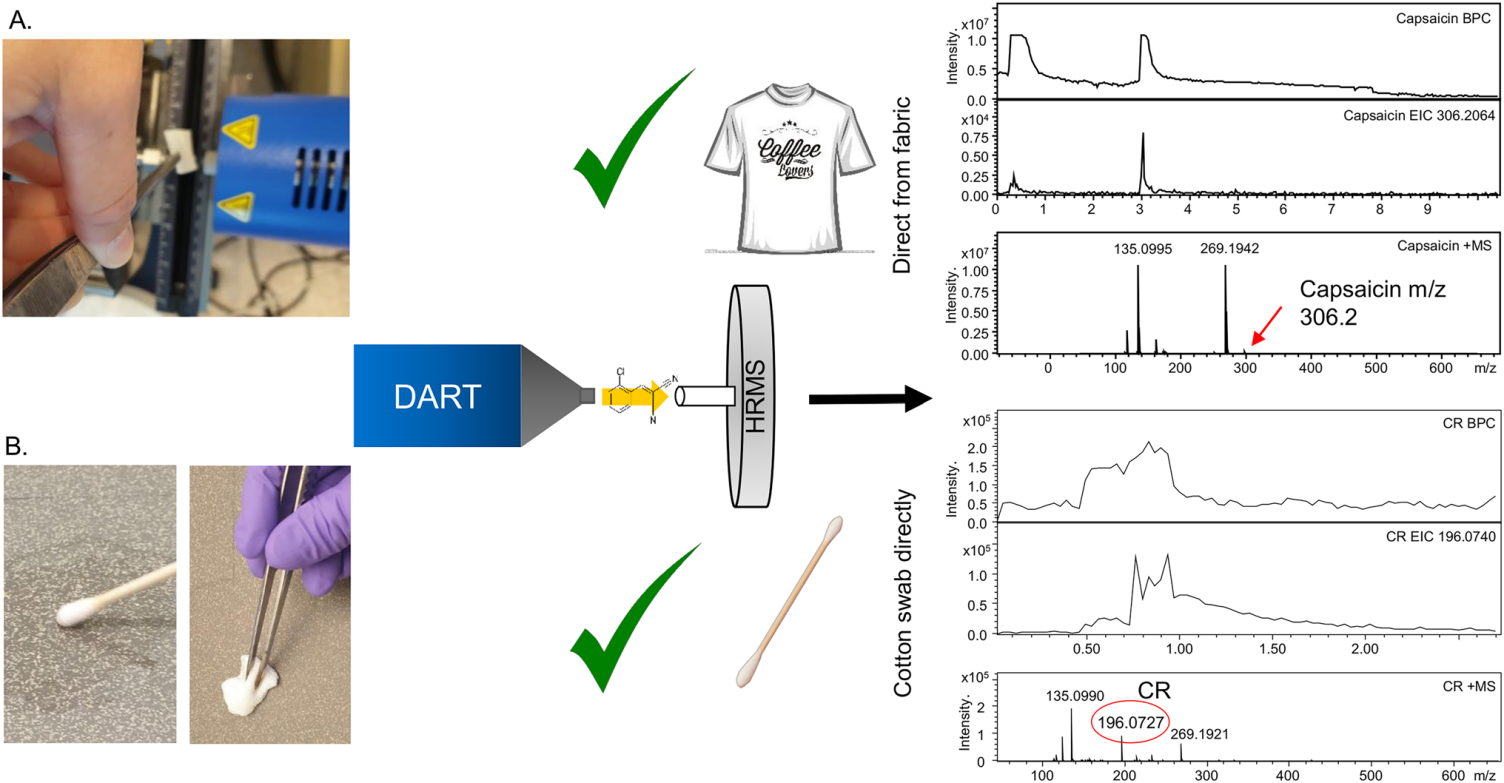
Schematic overview of the pilot study on surface sampling and direct analysis of samples by DART. **A** Surface sampling of fabric after treatment with two sprays, **B** Surface sampling of a ceramic floor tile using cotton wipes and swabs

To evaluate the feasibility of detecting the target agents on samples placed directly in the DART source without using the TD unit and sample traps, a pilot study was conducted in which surface samples were analysed using only the DART source and the HRMS instrument. To simulate the analysis of clothing, one OC and one CR spray were applied to pieces of cotton fabric that were then held directly in front of the DART source (see Fig. 3). Both active substances and the additives of each spray were successfully detected. Second, to simulate sampling of a suspected contaminated surface, the CR spray was applied to a ceramic floor tile and left to dry. The tile surface was then sampled using a cotton swab or a cotton wipe that was then held directly in front of the DART source. The active substance was successfully detected in both cases.

## Discussion

### Method development for detecting potential RCAs

To evaluate the viability of DART-TD-HRMS as a technique for fast, high throughput screening of RCAs, we developed a DART-TD-HRMS method that could detect every compound on the OPCW list [5]. The TD and DART temperatures were identified as the factors with the greatest influence on the detection of these compounds; the signal intensity for compounds belonging to the OC family increased strongly with the temperature and was highest at temperatures > 300°C, but such high temperatures adversely affected the detection of compounds such as CN and CS. It is possible that these compounds partially thermally degrades at such high temperatures. A compromise temperature of 300°C was therefore used in both the TD and the DART system. The two diimines (*N,N'*-bis(tert-butyl)ethylenediimine and *N,N'*-bis(isopropyl) ethylenediimine) did not ionise well under any conditions but by using a ten times higher amount they could be detected by the described method.

The method’s reproducibility and robustness differed between analytes, with compounds belonging to the OC family having the highest RSD values. It should be noted that this study did not aim to find optimal settings for individual compounds; if focusing exclusively on a single compound, it would be best to develop a new analytical method optimized for that compound alone. Despite this, our method achieved an LLOD of 5 ng for CN, CR, capsaicin and PAVA for with calibration curves was established. Notable, calibration curves were not established for all sixteen compounds since the focus was not quantitation but rather detection.

### Analysis of self-defence sprays on clothing

The practical utility of the developed method was evaluated by analysing samples of cotton fabric sprayed with various self-defence sprays. Some of these sprays were a few years past their stated expiry date, which may have affected the concentration of their active agents. These sprays were nevertheless included in the experiment on the grounds that if the method could detect the active agent in an old spray, it would certainly also be able to detect the higher concentrations in a new one.

Self-defence sprays contain large quantities of additives to solubilise the RCAs and facilitate their dispersal. While these additives complicate the analysis and detection of RCAs, their presence may also strongly suggest that an RCA has been used. When analysing the self-defence sprays, we observed several high abundance peaks that were identified as additives. These volatile additives produced very strong signals when analysing samples 1 hour after spraying and sometimes obscured the signal of the active substance, making these samples the most challenging to analyse. The volatile additives evaporated over time, so samples analysed with longer delays after spraying exhibited cleaner profiles. As a result, the active substance signals for some sprays were stronger in samples analysed 1 week after spraying than in those analysed 1 hour after spraying (Fig. 2). The problems caused by additives in recently sprayed samples can be alleviated by wiping the sprayed material with several sample traps in succession and analysing only the last one. However, this approach could not be used here because it was necessary to use the same sampling strategy for all time points for comparative purposes.

Hazmat teams routinely carry cotton swabs, cotton wipes, and scissors [31, 32]. However, they are not generally equipped with sample traps. Therefore, a pilot study was conducted to determine whether RCAs could be detected on surface samples using the DART source and detector without the TD unit or sample traps. To this end, pieces of sprayed cotton and samples collected by wiping cotton swabs or wipes over sprayed ceramic tiles were held in front of the DART source for analysis. In all cases, the RCAs and additives present in the sprays were successfully detected and no interfering ions were present in any of the samples analysed during the pilot study. However, this approach exhibited limited reproducibility and would require cutting of the clothing to be analysed in a real scenario. The success of these preliminary tests demonstrated the robustness of the instrumental setup and is notable because although thermal desorption generally increases reproducibility and facilitates analysis, thermal desorption modules may be unavailable or not functioning properly. It is therefore useful that identification is possible using only a DART source and a detector.

A significant difference between an LC system and the DART assembly is that the mass spectrometer quickly becomes dirty when using DART, which affects the instrument’s performance. This is unsurprising for several reasons. First, DART analysis introduces samples in line with the MS source, so everything in the sample (charged or not) enters the mass spectrometer. Conversely, an ESI source introduces samples at a 90° angle so only charged particles enter the mass spectrometer. This problem is particularly severe when analysing an object such as a piece of cotton or a swab that is held directly between the DART source and the mass spectrometer. Consequently, this approach greatly reduces the number of samples that can be analysed before cleaning when compared to protocols that use a TD module and sample traps. Second, the DART set-up can tolerate higher sample concentrations than LC-HRMS; while this is beneficial in several ways, it does cause dirt to accumulate in the instrument more rapidly than would be the case when using less concentrated samples in solution. The accumulation of dirt caused the sudden loss of low masses, making it easy to see when cleaning was required even though it was difficult to predict a priori how many samples could be analysed without cleaning. The hexapol cartridge in the mass spectrometer inlet had to be cleaned three times over a 3-month period during which almost 400 samples were analysed, meaning that around 130–150 samples were analysed between cleanings.

### Future perspective

DART-TD-HRMS is a fast technique with high sample throughput for detecting harmful substances. However, it relies on the use of advanced HRMS instruments maintained and operated by highly trained personnel such as the staff of a dedicated national laboratory. Consequently, samples collected in the field can only be analysed after transportation to a specialised facility.

Fieldable MS is of great interest in defence and forensic applications [33] and DART coupled to simple mass analysers have been deployed in field settings [26, 34, 35]. Other interesting field adaptable techniques suitable for hazardous compounds are CFI-APCI-ITMS [36]. Our long-term objective is to adapt the DART technique to field use, for instance by hazmat teams or in deployable CBRN laboratories. The study presented here represents a first step in this process. The next step will involve coupling the DART source to a smaller detector that can function under field conditions, for example, a small single quadrupole mass spectrometer. Despite its inferior mass resolution and sensitivity compared to an HRMS, a single quadrupole instrument has three key advantages: it is more robust, more cost-effective, and easier to operate and maintain [37]. This means that the operator requires little MS knowledge, making such instruments suitable for a wider range of laboratories.

## Conclusions

The main strengths of DART-TD-HRMS are its speed and high throughput, which make it suitable for rapidly analysing samples suspected to contain harmful compounds in cases where quick identification and response are vital and the number of samples are large. When connected to a TD module, DART-analysis are performed using sample traps onto which the analyte is loaded either by wiping a contaminated surface or by placing a droplet of an analyte solution directly on the trap. In both cases, the result of the analysis is obtained immediately after sampling. Several sample traps can be analysed in sequence, giving a high sample throughput. Our results show that this technique is well suited for analysing pure RCA compounds and identifying RCAs in self-defence sprays from surface samples up to 3 weeks after spray exposure. Furthermore, the technique could be adapted for field use by replacing the HRMS system with a simpler single quadrupole instrument for mass detection, potentially making DART-MS an accessible tool for a wide range of laboratories.

### Supplementary Information

Below is the link to the electronic supplementary material.

Supplementary file1 (DOCX 727 KB)
