# Supplementary material for: Rapid screening of riot control agents using DART-TD-HRMS
Source: Forensic Toxicol. 2024 Feb 22;42(2):152–62. doi: 10.1007/s11419-024-00681-5 (PMC11269514; doi:10.1007/s11419-024-00681-5)
Supplement: Supplementary file 1 — Supplementary file1 (DOCX 727 KB) [file 11419_2024_681_MOESM1_ESM.docx]

Rapid Screening of Riot Control Agents using DART-TD-HRMS

Lina Mörén, Anders Östin, Andreas Larsson, Julia Forsberg, Daniel Wiktelius, Pernilla Lindén*

# Supplementary Materials

## Data S1

### **General**

**All reactions were carried out under an inert atmosphere with dry solvents, unless otherwise stated. TLC was performed on Silica Gel 60 F254 using detection with UV light and staining with potassium permanganate or cerium molybdate. Automated flash column chromatography was performed using a Biotage® Isolera One system and purchased pre-packed silica gel cartridges (Biotage® SNAP Cartridge) The ^1^H and ^13^C NMR spectra were recorded at 298 K with a Bruker DRX-500 and calibrated using the residual peak of solvent as internal standard [CDCl_3_ (CHCl_3_ δH 7.26 ppm, CDCl_3_ δC 77.16 ppm), CD_3_CN (CD_2_HCN δH 1.94 ppm, CD_3_*C*N 118.7 ppm)]**

### **N-Vanillyl-9-methyldec-7-(E)-enamide (homocapsaicin)**

***i*-Pr_2_NEt (0.33 mL, 1.9 mmol) and Vanillylamine hydrochloride (100 mg, 0.5 mmol) was added to a solution of (*E*)-9-Methyl-7-decenoic acid [1] (115 mg, 0.6 mmol) in EtOAc:DMF 10:1 (5.5 mL) and the resulting solution was cooled to 0 ^o^C. Propanephosphonic acid anhydride (50% wt in EtOAc, 1.13 mL, 1.9 mmol) was added and the reaction was allowed to stir for 5 h. EtOAc (45 mL) and 1M HCl (25 mL) was added and the phases separated. The organic phase was washed with 1 M HCl (25 mL), NaHCO_3_ aq. sat. (2 x 25 mL) and brine (25 mL), dried over MgSO_4_, filtered and concentrated. The residue was purified with column chromatography on silica gel (1:10-3:1 EtOAc:heptane) to give 145 mg homocapsaicin as a clear oil (86% yield).**

**^1^H NMR (500 MHz, CDCl_3_):** δ **6.86 (d, *J* =8.1 Hz, 1H), 6.81 (d, *J*=1.8 Hz, 1H), 6.76 (dd, *J=*8.1, 1.8 Hz, 1H), 5.62 (br s, 1H), 5.59 (s, 1H), 5.39-5.28 (m, 2H), 4.35 (d, *J*=5.7 Hz, 2H), 3.88 (s, 3H), 2.26-2.16 (m, 3H), 1.96 (q, *J*=6.2 Hz, 2H), 1.65 (quint, *J*=7.5 Hz, 2H), 1.40-1.28 (m, 4H), 0.96 (d, *J*=6.7 Hz, 6H). ^13^C NMR (125 MHz, CDCl_3_):** δ 172.8, 146.7, 145.1, 137.8, 130.4, 126.8, 120.8, 114.3, 110.7, 55.9, 43.5, 36.8, 32.3, 31.0, 29.3, 28.8, 25.6, 22.6.

### N-Vanillyl-9-methyldecanamide (homodihydrocapsaicin)

Homodihydrocapsaicin was synthesized according to the same procedure as for homocapsaicin described above starting from 9-methyldecanoic acid [1] (116 mg, 0,6 mmol) and gave 147 mg (87% yield) of the title compound as a clear oil.

**^1^H NMR (500 MHz, CDCl_3_):** δ **6.86 (d, *J* =8.1 Hz, 1H), 6.81 (d, *J*=1.8 Hz, 1H), 6.76 (dd, *J=*8.1, 1.8 Hz, 1H), 5.62 (br s, 1H), 5.59 (s, 1H), 4.36 (d, *J*=5.7 Hz, 2H), 3.88 (s, 3H), 2.20 (t, *J*=7.5 Hz, 2H), 1.65 (quint, *J*=7.5 Hz, 2H), 1.50 (sept, *J*=6.7 Hz, 1H), 1.35-1.21 (m, 8H), 1.17-1.10 (m, 2H), 0.86 (d, *J*=6.7, 6H). ^13^C NMR (125 MHz, CDCl_3_):** δ **172.9, 146.7, 145.1, 130.4, 120.8, 114.3, 110.7, 55.9, 43.5, 39.0, 36.9, 29.8, 29.4, 29.3, 28.0, 27.3, 25.8, 22.7.**

### 4-Acetylaminodicyclohexylmethane

Acetyl chloride (25 µL, 0.35 mmol) was added to a solution of 4-Aminodicyclohexylmethane ^[1]^ (34 mg, 0.17 mmol) and *i*-Pr_2_NEt (61 µL, 0.35 mmol) in CH_2_Cl_2_ (0.5 mL) at 0 ^o^C. The reaction was allowed to warmed to RT and stirred for 4 h, before quenched with 1 M HCl (3 mL). The mixture was extracted with CH_2_Cl_2_ (3 x 3 mL) and the combined organic phases was washed with brine, dried over Na_2_SO_4_, filtered and concentrated. The residue was purified with **automated flash column chromatography with a 5-100% gradient of EtOAc in Heptane to give 40 mg of 4-Acetylaminodicyclohexylmethane (97% yield, mixture of stereoisomers) as a white solid.**

**^1^H NMR (500 MHz, CDCl_3_):** δ **5.56-5.18 (m, 1H), 4.05-3.61 (m, 1H), 2.01-1.91 (m, 6H), 1.76-1.51 (m, 11H), 1.33-0.76 (m, 11H).**

### N,N'-Bis(isopropyl)ethylenediimine

Glyoxal (2.9 mL, 40% wt in H_2_O, 25 mmol) was added dropwise to isopropylamine (4.3 mL, 50 mmol) at 0 ^o^C. The formed solid material was allowed to warm to RT and then heated with a heat-gun to obtain a two-layered solution. The top layer was isolated and allowed to solidify. The crude product was recrystallized from Et_2_O to give 2.05 g of N,N'-Bis(isopropyl)ethylenediimine (58% yield)

**^1^H NMR (500 MHz, CD_3_CN):** δ 7.86 (s, 2H), 3.49 (sept, *J*=6.3 Hz, 2H), 1.16 (d, *J*=6.3 Hz, 12H). **^13^C NMR (125 MHz, CD_3_CN):** δ 161.0, 62.3, 24.5.

Nordihydrocapsaicin and *N,N'*-bis(tert-butyl)ethylenediimine was synthesized as previously described [1]. Dibenzoxazepine (CR) was synthesized according to a previously published procedure [2]. 2-chlorobenzalmalononitrile (CS) was synthesized according to a previously published procedure [3].


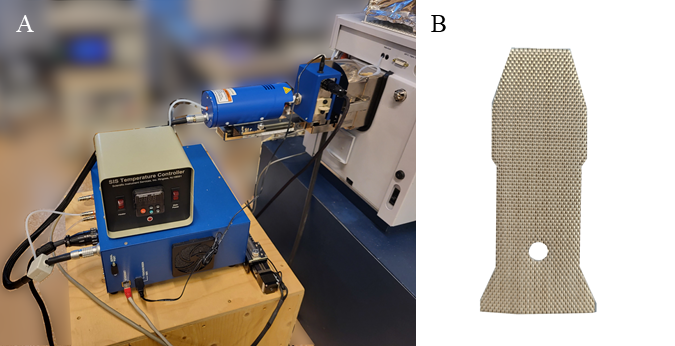


**Fig S1** A) DART-TD-HRMS setup. B) Sample trap.


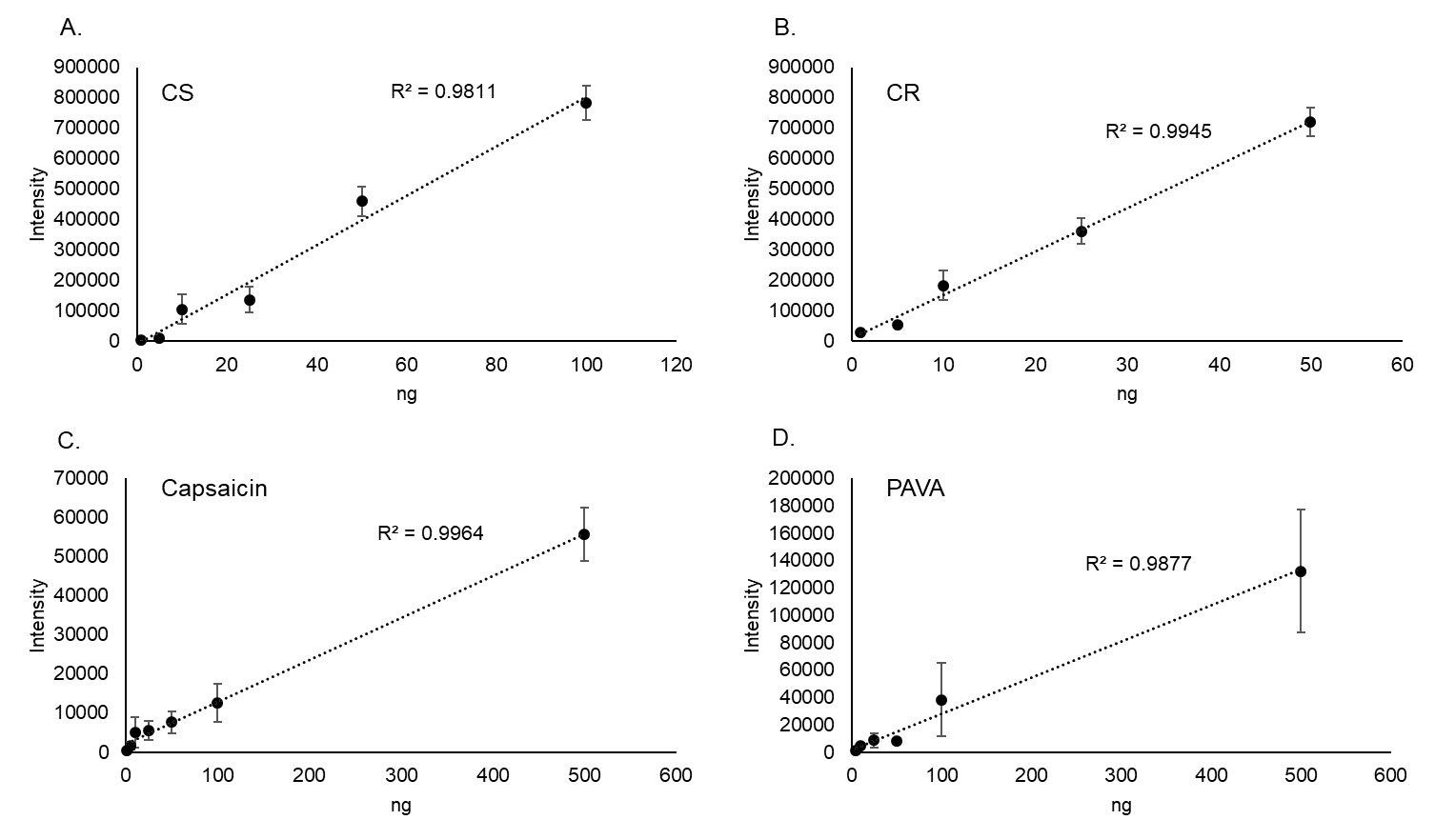


**Fig S2** Concentration curves showing the linear concentration ranges for four common active substances in self-defence sprays analysed by DART-TD-HRMS. A) CS (1, 5, 10, 25, 50, 100 ng), B) CR (1, 5, 10, 25, 50 ng), C) capsaicin (1, 5, 10, 25, 50, 100, 500 ng) and D) PAVA (1, 5, 10, 25, 50, 100, 500 ng). Each data point is an average of three measurements with error bars showing the standard deviation.


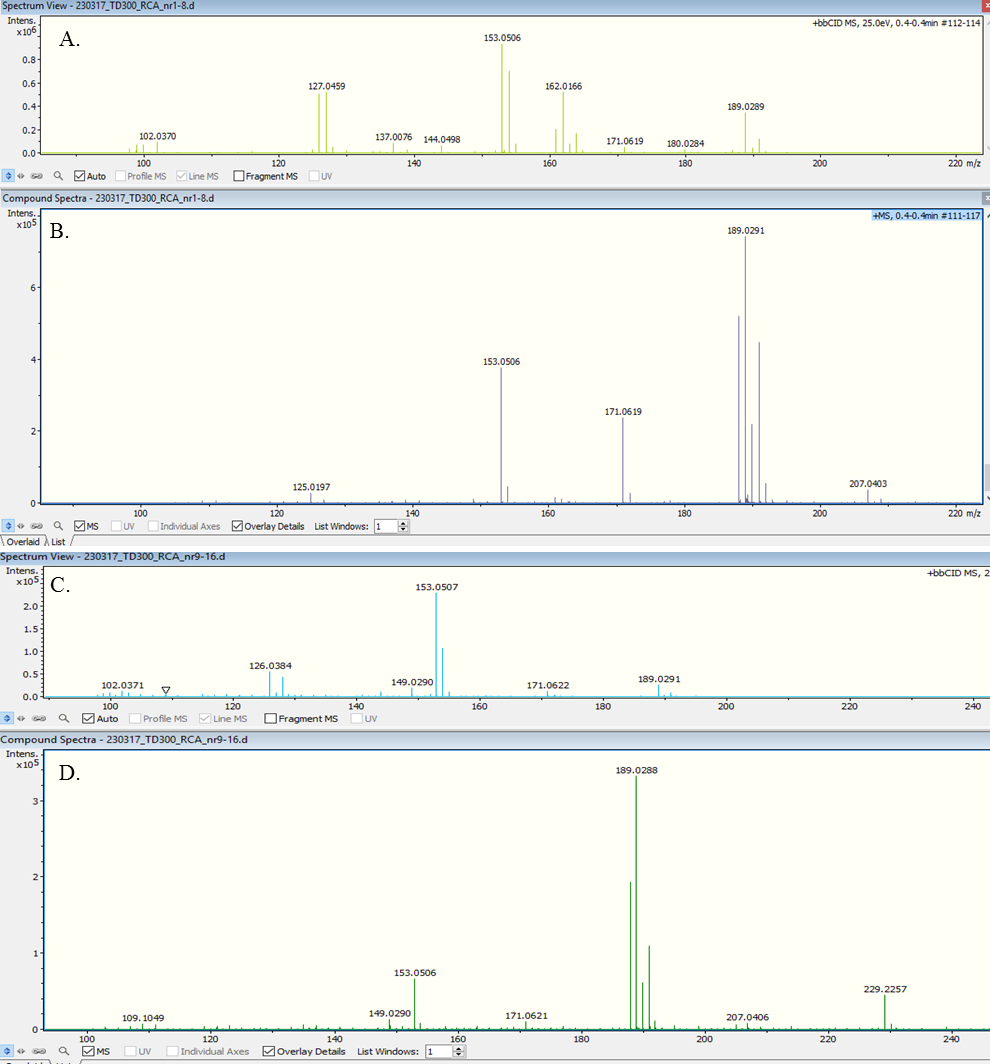


**Fig S3** A) bbCID CS, B) All MS CS, C) bbCID α-chlorobenzylidenemalononitrile, D) All MS α-chlorobenzylidenemalononitrile.

**Table S1** List of in-house reference collection of self-defence sprays included in the study stating expiry data, stated active substance and additives.

| **ID** | **Expiry date** | **Stated Active Substance** | **Stated Additives** |
| --- | --- | --- | --- |
| Spray 1 | Oct 2021 | OC- pseudocapsaicin | IPA, propylene glycol |
| Spray 2 | Dec 2020 | OC- capsaicin | IPA, dipropylene glycol |
| Spray 3 | Nov 2018 | OC- capsaicin | IPA, dipropylene glycol |
| Spray 4 | Nov 2018 | CS | IPA, dipropylene glycol |
| Spray 5 | Nov 2018 | CS | IPA, dipropylene glycol |
| Spray 6 | Nov 2018 | OC- capsaicin | IPA, dipropylene glycol |
| Spray 7 | Mar 2021 | OC- pseudocapsaicin | IPA, propylene glycol |
| Spray 8 | Jul 2020 | OC- pseudocapsaicin | IPA, propylene glycol |
| Spray 9 | Unreadable | OC- capsaicin | IPA, dipropylene glycol |
| Spray 10 | Nov 2018 | CS | IPA, dipropylene glycol |
| Spray 11 | Dec 2021 | OC- capsaicin | ethanol, IPA, glycerol |
| Spray 12 | May 2018 | OC- capsaicin | not stated |
| Spray 13 | Dec 2021 | CS | not stated |
| Spray 14 | Oct 2021 | OC- pseudocapsaicin | IPA, propylene glycol |
| Spray 15 | Dec 2022 | OC- capsaicin | IPA, dipropylene glycol |
| Spray 16 | Dec 2021 | OC- capsaicin | IPA, dipropylene glycol |
| Spray 17 | Jun 2022 | OC- capsaicin | IPA, dipropylene glycol |
| Spray 18 | Dec 2021 | OC- capsaicin | IPA, dipropylene glycol |
| Spray 19 | Jun 2022 | OC-pseudocapsaicin | propylene glycol |
| Spray 20 | Jun 2022 | CS | IPA |

**Table S2.** Reproducibility and robustness values for analyses of the 16 OPCW-listed RCAs using the developed method.

|  |  | **Reproducibility** | | **Robustness** | |
| --- | --- | --- | --- | --- | --- |
| **Compound** | **[M+H]+** | **Amount on sample trap (ng)** | **RSD%** | **Amount on sample trap (ng)** | **RSD%** |
| CN | 155.0258 | 50 | 11 | 50 | 52 |
| CS | 189.0214 | 50 | 10 | 50 | 0.1 |
| CR | 196.0757 | 50 | 16 | 50 | 0.2 |
| homocapsaicin | 320.2220 | 50 | 27 | 50 | 50 |
| homodihydrocapsaicin | 322.2377 | 50 | 29 | 50 | 68 |
| 4-nonanoylmorpholine | 228.1958 | 50 | 14 | 50 | 19 |
| 3’-chloroacetophenone | 155.0258 | 50 | 17 | 50 | 41 |
| α-chlorobenzylidenemalononitrile | 189.0214 | 50 | 17 | 50 | 7 |
| capsaicin | 306.2064 | 100 | 29 | 50 | 26 |
| dihydrocapsaicin | 308.2220 | 100 | 33 | 50 | 77 |
| PAVA | 294.2064 | 100 | 10 | 50 | 59 |
| nordihydrocapsaicin | 294.2064 | 100 | 38 | 50 | 108 |
| 2’-chloroacetophenone | 155.0258 | 100 | 21 | 50 | 37 |
| cis-4-acetylaminodicyclohexylmethane | 238.2165 | 100 | 12 | 50 | 3 |
| N,N'-bis(isopropyl)ethylenediimine | 141.1386 | 500 | 23 | 50 | 34 |
| N,N'-bis(tert-butyl)ethylenediimine | 169.1699 | 500 | 27 | 50 | 113 |

Reproducibility is quantified in terms of the relative standard deviation **(**RSD%) for seven sequential measurements of each analyte. Robustness was determined with a fixed loading of 50 ng (CS and CR are saturated).

**Table S3.** Stated and detected additives in twenty self-defence sprays analysed by DART-TD-HRMS. Each spray was applied to pieces of cotton fabric that were then sampled using sample traps one hour after spraying.

| ID | Active Substance | Stated  Additives | Dipropylene glycol [M+H]^+^ (135.1016) | Dipropylene glycol [M2+H]^+^ (269.1959) | Unknown C_6_H_10_O_2_ [M+H]^+^ (115.0772) | Butyl acetate [M+H]^+^ (117.091) | Butyl diglycol [M+H]^+^ (163.1329) | Unknown C_7_H_12_N_4_ [M+H]^+^ (153.1135) |
| --- | --- | --- | --- | --- | --- | --- | --- | --- |
| Spray 1 | OC | IPA, propylene glycol |  |  |  |  |  |  |
| Spray 2 | OC | IPA, dipropylene glycol | x | x | x | x | x |  |
| Spray 3 | OC | IPA, dipropylene glycol | x | x | x | x | x | x |
| Spray 4 | CS | IPA, dipropylene glycol | x |  |  | x | x |  |
| Spray 5 | CS | IPA, dipropylene glycol | x |  | x | x |  | x |
| Spray 6 | OC | IPA, dipropylene glycol | x | x | x | x | x | x |
| Spray 7 | OC | IPA, propylene glycol |  |  |  |  |  |  |
| Spray 8 | OC | IPA, propylene glycol |  |  |  |  |  | x |
| Spray 9 | OC | IPA, dipropylene glycol | x | x | x | x | x | x |
| Spray 10 | CS | IPA, dipropylene glycol |  |  |  |  |  |  |
| Spray 11 | OC | IPA, Ethanol, Glycerol |  |  |  |  |  |  |
| Spray 12 | OC | Not stated | x | x | x | x | x | x |
| Spray 13 | CS | Not stated | x | x |  | x |  |  |
| Spray 14 | OC | IPA, propylene glycol | x | x | x | x |  |  |
| Spray 15 | OC | IPA, dipropylene glycol | x | x | x | x |  |  |
| Spray 16 | OC | IPA, dipropylene glycol | x | x | x | x | x | x |
| Spray 17 | OC | IPA, dipropylene glycol | x | x | x | x | x | x |
| Spray 18 | OC | IPA, dipropylene glycol | x | x | x | x | x | x |
| Spray 19 | OC | propylene glycol | x | x | x | x | x | x |
| Spray 20 | CS | IPA |  |  |  |  |  |  |

The “x” denotes if an additive has been detected in the spray.

## References

1. Wiktelius, D., J. Forsberg, and A. Östin, *Syntes av inkapaciterande ämnen- ovanliga komponenter i tårgaser och pepparsprayer.* FOI MEMO 5823, 2016: p. Swedish Defence Research Agency.

2. Wardrop, A.W.H., et al., *Preparation of some dibenz[b,f][1,4]oxazepines and dibenz[b,e]azepines.* J. Chem. Soc., Perkin Trans. 1, 1976(12): p. 1279-1285.

3. Rosin, J., *Production of o-chlorobenzamalononitrile, United States, US3549683A 1970-12-22*.
